# Alternative stable states of microbiome structure and soil ecosystem functions

**DOI:** 10.1186/s40793-025-00688-4

**Published:** 2025-03-06

**Authors:** Hiroaki Fujita, Shigenobu Yoshida, Kenta Suzuki, Hirokazu Toju

**Affiliations:** 1 Laboratory of Ecosystems and Coevolution, Graduate School of Biostudies, Kyoto University, Kyoto, 606-8501 Japan; 2 Center for Living Systems Information Science (CeLiSIS), Graduate School of Biostudies, Kyoto University, Kyoto, 606-8501 Japan; 3 Institute for Plant Protection, National Agriculture and Food Research Organization, Tsukuba, 305-8666 Ibaraki Japan; 4 Integrated Bioresource Information Division, BioResource Research Center, RIKEN, Tsukuba, 305-0074 Ibaraki Japan; 5 Institute of Multidisciplinary Sciences, Yokohama National University, Yokohama, 240-8501 Kanagawa Japan

## Abstract

**Background:**

Theory predicts that biological communities can have multiple stable states in terms of their species/taxonomic compositions. The presence of such alternative stable states has been examined in classic ecological studies on the communities of macro-organisms (e.g., distinction between forest and savanna vegetation types). Nonetheless, it remains an essential challenge to extend the target of the discussion on multistability from macro-organismal systems to highly species-rich microbial systems. Identifying alternative stable states of taxonomically diverse microbial communities is a crucial step for predicting and controlling microbiome processes in light of classic ecological studies on community stability.

**Results:**

By targeting soil microbiomes, we inferred the stability landscapes of community structure based on a mathematical framework of statistical physics. We compiled a dataset involving 11 archaeal, 332 bacterial, and 240 fungal families detected from > 1,500 agroecosystem soil samples and applied the energy landscape analysis to estimate the stability/instability of observed taxonomic compositions. The statistical analysis suggested that both prokaryotic and fungal community structure could be classified into several stable states. We also found that the inferred alternative stable states differed greatly in their associations with crop disease prevalence in agroecosystems. We further inferred “tipping points”, through which transitions between alternative stable states could occur.

**Conclusion:**

Our results suggest that the structure of complex soil microbiomes can be categorized into alternative stable states, which potentially differ in ecosystem-level functioning. Such insights into the relationship between structure, stability, and functions of ecological communities will provide a basis for ecosystem restoration and the sustainable management of agroecosystems.

**Supplementary Information:**

The online version contains supplementary material available at 10.1186/s40793-025-00688-4.

## Background

Since the late 1960s, theoretical ecologists have discussed that biological community compositions could have multiple stable states [1–3]. The concept of “multistability” has been examined in aquatic and terrestrial ecosystems, mainly targeting non-microbial communities [4–6]. Community structure in shallow lakes, for example, is known to show two discrete states depending on nutrient (phosphorus) concentrations as represented by the bistability of charophyte densities [2, 7, 8]. Likewise, worldwide inventories of vegetation have shown the lack of intermediate states between forest (tree cover = ca. 80%) and savanna (tree cover = ca. 20%), indicating the presence of alternative stable states [9–11]. Importantly, these discrete vegetational types in each of the aquatic (high vs. low charophyte densities) and terrestrial (forest vs. savanna) ecosystems differ greatly in ecosystem-scale productivity and processes (e.g., energy flow through food webs) [7–11]. Thus, alternative stable states of biological community structure can critically affect ecosystem-scale properties not only in natural ecosystems but also in ecosystems managed by humans (e.g., fishery, agricultural, and forestry production) [2, 4, 12, 13]. Nonetheless, we still have limited knowledge of the relationship among the structure, stability, and functions of communities that composed of taxonomically diverse microbes.

While classic studies targeting freshwater and terrestrial biomes have explored community multistability based on simple characterization of community states (e.g., tree cover percentages), recent technical advances in microbial community (microbiome) research have come to provide general statistical frameworks for systematically evaluating biological community stability [14–19]. In recent years, large datasets of microbial species/taxonomic compositions have been made available with the aid of amplicon and shotgun sequencing technologies, providing a basis for exploring reproducible states in microbiome community structure [17, 20, 21]. Such high-throughput DNA sequencing studies in medicine, for example, have shown that human individuals can be classified into a few semi-discrete clusters in terms of their intestinal microbiome compositions [22–25]. Intriguingly, these alternative gut microbiomes (“enterotypes”) differ in their associations with human disease such as type II diabetes and Crohn’s disease [16]. In addition to those studies on human-associated microbiomes [22, 26, 27], studies on plant-associated microbiomes have started to reorganize our recognition of how multistability of phyllosphere/rhizosphere microbiome structure is associated with ecosystem-scale processes and functions [15, 28]. Because hundreds or thousands of community samples (i.e., samples from > 1,000 human individuals) are available in such animal- or plant-associated microbiome studies, it is now possible to discuss the potential relationship between community structure and ecosystem functions based on statistical signs of the presence of alternative stable states.

**Fig. 1:**
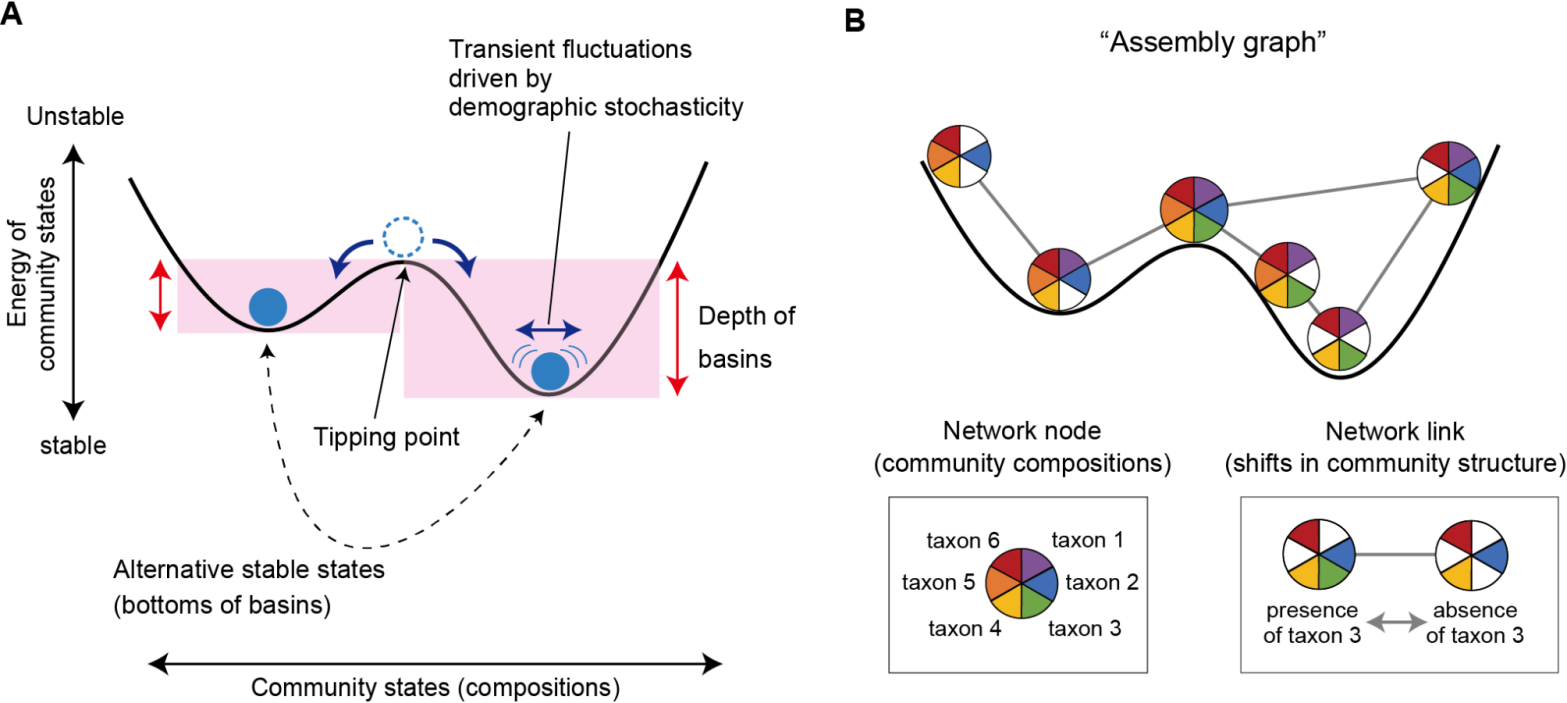
Schema of multistability of ecological communities. (**A**) Alternative stable states and tipping points. The structure of “stability landscapes” showing relationship between community states (species or taxonomic compositions) and their stability is inferred based on the energy landscape analysis. The “energy” of each community state is calculated with maximum entropy models as detailed in Methods. Lower energy represents a more stable community state on a stability landscape. Transient fluctuations around alternative stable states (i.e., attractors or bottoms of basins) are assumed as probabilistic phenomena in the statistical approach. (**B**) Assembly graph. To explore numerous possible states of real ecological communities, input data are binarized in the energy landscape analysis. Potential transitions between community states are then considered within “assembly graphs”, in which paths between different species/taxonomic compositions are treated as network links. Thus, by the assembly-graph approach, the energy landscape analysis provides a general framework for inferring the structure of stability landscapes in empirical studies of complex microbiome datasets

In theoretical ecology, stability of community states (taxonomic or species compositions) is often discussed in the framework of stability landscapes [3, 4, 29–31]. On the landscape representing stability/instability of community structure, alternative stable states (i.e., the bottoms of the “basins of attraction”) are split by “tipping points” representing unstable equilibria [3, 4, 12, 29] (Fig. 1). As these stable states differ in the biological functions of constituent communities, stable and highly functional community states can be explored within the stability landscapes. With the application of a recently proposed mathematical approach developed based on statistical physics [32, 33], it is now possible to infer the structure of stability landscapes based on empirical datasets of ecological communities [29, 34, 35]. The statistical framework called “energy landscape analysis” explore the probabilities of community compositions within the “assembly graphs” [36, 37], which represent paths of possible community assembly [20, 29] (Fig. 1). Although hundreds or thousands of community compositional data points are required to apply the statistical approach [20, 29], the energy landscape analysis offers a powerful way to identify key features of stable and highly functional microbiome states out of numerous possible combinations of microbial species or taxa. Despite the potential for systematically profiling key community-scale properties based on massive datasets, the energy landscape analysis has been applied only to a few microbial community datasets [20, 29].

We here apply the emerging statistical framework to soil microbiomes, which often show highest levels of structural diversity in nature. We compile a cropland soil microbiome dataset consisting of > 1,500 sampling positions across the Japan Archipelago [38]. With the massive dataset, we evaluate the compositional stability of prokaryotic and fungal communities based on the maximum entropy models of the energy landscape analysis [29]. We then examine whether the inferred alternative stable states of soil microbiomes can differ in their associations with crop disease prevalence. We also identify key microbial taxa whose abundance can be used to define alternative stable states with favorable and unfavorable ecosystem functions. The results of the energy landscape analysis are further used to infer tipping points splitting the basins of inferred alternative stable states. Overall, this study illustrates how we can integrate the information of community structure, stability, and functions based on a statistical platform commonly applicable to diverse microbial and macro-organismal communities. Such insights will help us build practical frameworks for shifting ecological communities from unfavorable to favorable states in the contexts of conservation biology and sustainable agriculture.

## Methods

### Dataset compilation

**Fig. 2:**
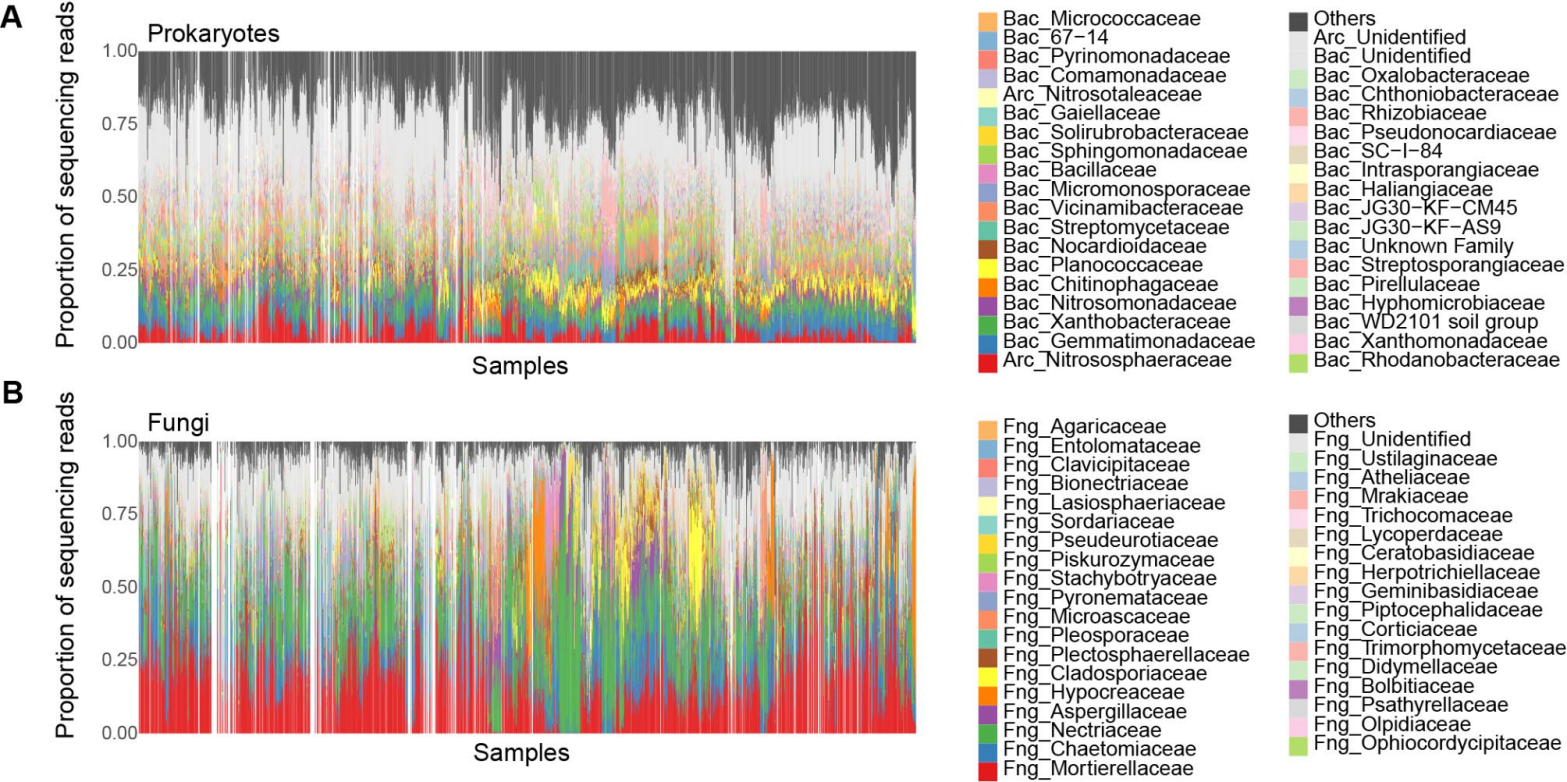
Community structure of the source data. The family-level compositions of prokaryotes (**A**) and fungi (**B**) are shown based on the source dataset [38]. The soil samples from which DNA sequence data were unavailable for either prokaryotic 16 S rRNA or fungal ITS regions are indicated as blanks. The percentages of the variance explained by the PCoA1 or PCoA2 axis of community structure are shown in parentheses. See Additional file 1: Fig. S1 for community compositions at the order and genus levels

We compiled a publicly available dataset of cropland soil microbiomes (DDBJ accession: DRA015491; Fig. 2) with its metadata of the samples [38]. In the previous study reporting the data [38], 2,903 bulk soil samples collected from the field of 19 crop plant species (apple, broccoli, cabbage, celery, Chinese cabbage, eggplant, ginger, komatsuna, lettuce, onion, potato, radish, rice, satsuma mandarin, soybean, spinach, strawberry, sweet corn, tomato) across the Japan Archipelago from January 23, 2006 to July 28, 2014 (latitudes of the sampling positions: 26.1–42.8 °N) were subjected to the amplicon sequencing analysis (i.e., sequencing of PCR-amplified DNA fragments) of the prokaryotic 16 S rRNA region and the fungal internal transcribed spacer 1 (ITS1) region [38]. The information of dry soil pH, electrical conductivity, carbon/nitrogen (C/N) ratio, and available phosphorous concentration was available for 2,830, 2,610, 2,346, and 2,249 samples, respectively. Likewise, the information of crop plant disease (the percentage of diseased plants or disease severity index [39]) was available for 1,471 samples [38]. The plant pathogens surveyed were *Colletotrichum gloeosporioides* on the strawberry, *Fusarium oxysporum* on the celery, the lettuce, the strawberry, and the tomato, *Phytophthora sojae* on the soybean, *Plasmodiophora brassicae* on Cruciferae plants, *Pyrenochaeta lycopersici* on the tomato, *Pythium myriotylum* on the ginger, *Ralstonia solanacearum* on the eggplant and the tomato, and *Verticillium* spp. on Chinese cabbage [38]. After a series of quality filtering, prokaryotic and fungal community data were available for 2,318 and 2,186 samples, respectively. In total, 579 archaeal amplicon sequence variants (ASVs) representing 11 families, 26,640 bacterial ASVs representing 332 families, and 6,306 fungal ASVs representing 240 families were detected [38] (Fig. 2; Additional file 1: Fig. S1).

### Community structure along environmental gradients

We first inspected how prokaryotic and fungal community structure varied along environmental gradients. For each data matrix representing the family-level compositions of prokaryotes or fungi, a principal coordinate analysis (PCoA) was performed based on Bray-Curtis *β*-diversity. The PCoA1 and PCoA2 scores were then plotted, respectively, along the gradient of each soil environmental factor. In total, 1,771 and 1,664 samples for which the information of both community structure and all the four environmental variables was available were included in the analyses of prokaryotes and fungi, respectively. For each plot representing relationship between community structure and a soil environmental variable, the density of data points was visualized with the ggplot2 3.3.6 package [40] of R v.4.1.2 [41].

### Energy landscape analysis

We examined the stability landscape of soil microbiome structure based on the framework of an energy landscape analysis [20, 29, 32] (tutorials and codes of energy landscape analyses are available at https://github.com/kecosz/rELA). In the framework, the term “energy” is defined by the following equations based on an approach of statistical physics [29, 32]. Within the “assembly graphs” representing paths of community dynamics [36, 37], probabilities of observing specific community compositions can be explored as detailed previously [29]. In brief, probabilities of community states $\:\text{p}\left({\overrightarrow{\sigma\:}}^{\left(k\right)}\right)\:$ are given by

1 $$\:P\left({\overrightarrow{\sigma\:}}^{\left(k\right)}|\epsilon\:\right)={e}^{-E({\overrightarrow{\sigma\:}}^{\left(k\right)},\epsilon\:)}/Z$$

2 $$\:Z={\sum\:}_{i=1}^{{2}^{S}}{e}^{-E({\overrightarrow{\sigma\:}}^{\left(i\right)},\epsilon\:)},$$

where $\:{\overrightarrow{\sigma\:}}^{\left(k\right)}=({\sigma\:}_{1}^{\left(k\right)},{\sigma\:}_{2}^{\left(k\right)},\:\dots\:,{\sigma\:}_{S}^{\left(k\right)})$ is a community state vector of *k*-th community state and *S* is the total number of taxa (e.g., ASVs, species, genera, or families) examined. $\:\epsilon\:=({\epsilon\:}_{1},{\epsilon\:}_{2},\:\dots\:,{\epsilon\:}_{M})$ is an array of continuous values representing environmental factors (e.g., soil pH and electrical conductivity) and *M* is the total number of environmental parameters. $\:{\sigma\:}_{\text{i}}^{\left(k\right)}$ is a binary variable that represents presence (1) or absence (0) of taxon *i*: i.e., there are a total of $\:{2}^{S}$ community states. As the exploration of the $\:{2}^{S}$ community states were computationally intensive, we coded community states at the family-level taxonomic compositions. Specifically, for each sample, families whose relative abundance exceeds a certain threshold value (threshold for binarization) were coded as 1, while the remaining minor families were coded as 0. Subsequently, families whose occurrence ratios (i.e., the proportions of samples in which target families were coded as 1) were less than a certain threshold (occurrence threshold) were excluded from the dataset. Likewise, families that appeared in almost all samples (1– occurrence threshold) were excluded. Note that without such thinning of input data, the dimensions of community states are too high to be explored even using supercomputers. Therefore, exclusion of the taxa that contribute little to the classification of community states (i.e., taxa appearing only in a small fraction of samples or those appearing in most samples) is inevitable in the energy landscape analysis. Through intensive preliminary computational runs with various combinations of binarization and occurrence thresholds, we found that the number of taxa (*S*) should be kept less than 65 as detailed in the next subsection.

When input community matrix is defined, the energy of the community state $\:{\overrightarrow{\sigma\:}}^{\left(k\right)}$ is given by the extended pairwise maximum entropy model:

3 $$\begin{array}{l}\:E({\overrightarrow {\sigma \:} ^{\left( k \right)}},\epsilon \:) = \: - \sum \: _{i = 1}^S{h_i}\overrightarrow {\sigma \:} _i^{\left( k \right)} - \sum {\:_{j = 1}^S} \sum {\:_{i = 1}^M} {g_{ij}}\epsilon \:_i^{\left( k \right)}\sigma \:_j^{\left( k \right)}\\\\- \sum \: _{i = 1}^S\sum \: _{j = 1,\:i \ne \:j}^S{J_{ij}}\overrightarrow {\sigma \:} _i^{\left( k \right)}\overrightarrow {\sigma \:} _j^{\left( k \right)}/2,\end{array}$$

where $\:{h}_{i}$ represents the net effect of implicit abiotic factors, by which *i*-th taxon is more likely to present (*h*_*i*_ > 0) or not (*h*_*i*_ < 0), $\:{g}_{ij}$ represents the effect of the *i-*th observed environmental factor, and $\:{J}_{ij}$ represents a co-occurrence pattern of *i*-th and *j*-th taxa. Since the logarithm of the probability of a community state is inversely proportional to $\:E\left({\overrightarrow{\sigma\:}}^{\left(k\right)}\right)$, a community state having lower *E* is more frequently observed. To consider dynamics on an assembly graph defined as a network whose $\:{2}^{S}$ nodes represent possible community states and the edges represents transition path between them (two community states are adjacent only if they have the opposite presence/absence status for just one species), we assigned energy to nodes with the above equation, and so imposed the directionality in state transitions. Then, by using the steepest descent algorithm [29], we identified nodes having the lowest energy compared to all its neighbors within the weighted network, and determined their basins of attraction [29, 31]. These community states whose energy was lower than that of all adjacent community states represent alternative stable states (attractors), around which community states are expected to show transient fluctuations due to demographic stochasticity as considered in the statistical framework [20, 29] (Fig. 1). Soil pH, electrical conductivity, C/N ratio, and available phosphorous concentration were included as environmental variables in the model after normalization within the ranges from 0 to 1.

### Energy landscape structure

The energy landscapes of community structure were inferred, respectively, for three types of datasets, namely, the prokaryotic community matrix, the fungal matrix, and the matrix including both prokaryotes and fungi. As mentioned above, various combinations of binarization and occurrence thresholds were examined to check the reproducibility of the results. In addition to the energy landscape analysis based on the above-mentioned family-level delineation of community states, analyses based on community-state delineation at the order-level were performed. In the main body and supplementary figures of this study, we show the results at the following settings: prokaryotes (family), binarization = 0.020, occurrence = 0.10; prokaryotes (order), binarization = 0.020, occurrence = 0.10; fungi (family), binarization = 0.001, occurrence = 0.05; fungi (family), binarization = 0.001, occurrence = 0.10; prokaryotes + fungi (family), binarization = 0.030, occurrence = 0.10; prokaryotes + fungi (order), binarization = 0.030, occurrence = 0.10. The thresholds were set to keep the dimensions of the state space within computationally feasible ranges (*S* < 65) as mentioned above.

For each setting, the parameters of the extended pairwise maximum entropy model [Eq. 3] were adjusted to the empirical data. More precisely, the maximum likelihood estimates of *h*_*i*_, $\:{g}_{ij}$, and $\:{J}_{ij}$ was obtained by a stochastic approximation method as detailed elsewhere [29]. The parameters were regularized by a logistic prior with location 0 and scale 2.0 (for environmental responses) or 0.5 (for pairwise relationships) [42]. Hyperparameters for the algorithm, criterion value for judging the convergence of parameters qth = 10^− 5^, were set according to a series of preliminary analyses. Based on the inferred maximum entropy model, we determined basins of attraction [31] within the energy landscape based on a steepest descent procedure [29]. The structure of the energy landscape was visualized by showing the energy of each soil sample on the two-dimensional surface of the community state space defined with the abovementioned PCoA scores. The default setting of environmental variables (the mean value for each of soil pH, electrical conductivity, C/N ratio, and available phosphorous concentration) was used in the energy calculation. Spline smoothing of the energy landscape was performed with optimized penalty scores using the mgcv v.1.8–40 package [43] of R. For each analysis of the prokaryote, fungi, and prokaryote + fungi datasets, 1,771, 1,664, and 1,474 samples for which the information of both community structure and all the four environmental variables was available were subjected to the analysis, respectively.

### Associations with crop disease level

For the inferred basins of microbial community compositions, associations with crop disease prevalence were examined. We first constructed the list of soil samples whose community structure was located within each basin of attraction. We then evaluated the ecosystem-scale properties of the basins using the metadata of crop disease symptoms [38]. Specifically, for each basin, we calculated the proportion of constituent soil samples with the lowest level of crop disease symptoms as defined by the following conditions: the percentage of diseased plants < 20 or disease severity index < 20 [38]. The alternative stable states representing different levels of crop disease prevalence were then compared in terms of taxonomic compositions in order to explore microbial taxa that were keys to distinguish potentially disease-suppressive and disease-promotive soil ecosystems.

### Disconnectivity graphs

For the reconstructed energy landscape, we inferred “disconnectivity graphs” [29] representing how basins of attraction were split by tipping points (Fig. 1A). Within a disconnectivity graph, alternative stable states whose energy is much lower than the energy of connected tipping points are expected to be resistant to perturbations (demographic stochasticity). In contrast, community states with small energy gaps to tipping points may be shifted from current basins to adjacent basins with even small perturbations.

## Results

### Community structure along environmental gradients

**Fig. 3:**
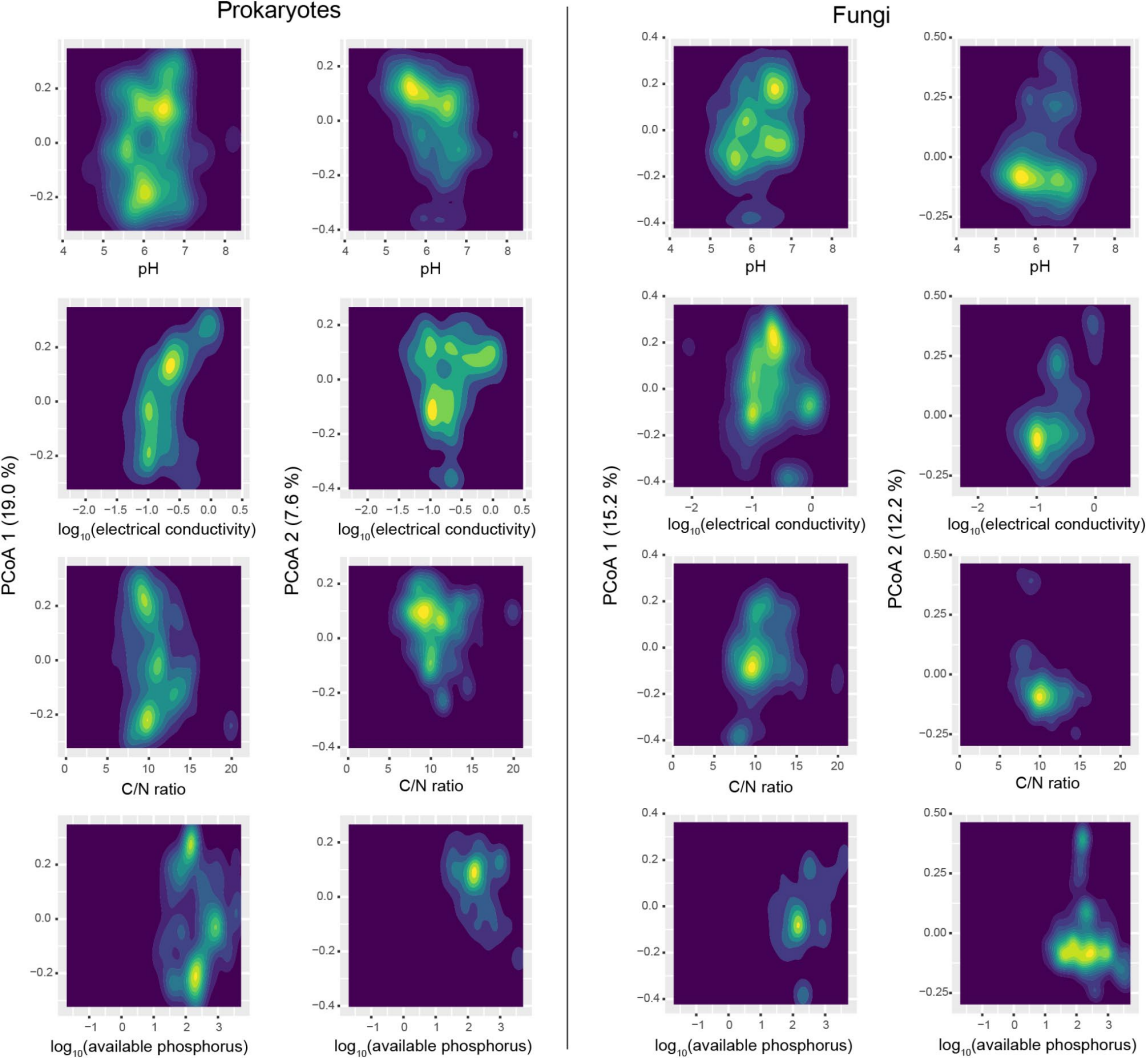
Community structure along environmental gradients. The scores representing prokaryotic/fungal community compositions (PCoA1 and PCoA2 scores) are shown along each of the soil environmental factors (pH, electrical conductivity, C/N ratio, and available phosphorous concentration). The density plots representing 1,771 prokaryotic (left) and 1,664 fungal community samples are shown. See Additional files 2–3: Figs. S2-3 for full the scatter plots showing the data points

On each plot showing community compositions (PCoA1 or PCoA2 scores) along a soil environmental gradient (Fig. 3), multiple clusters of data points were observed for both prokaryotes and fungi (Additional files 2–3: Figs. S2-3). For example, two high-density regions of prokaryotic community data points were observed for the PCoA1 axis when pH = 6.0 (Fig. 3). Likewise, fungal community PCoA1 scores showed a bimodal pattern around pH = 6.7 (Fig. 3).

### Energy landscape structure

**Fig. 4:**
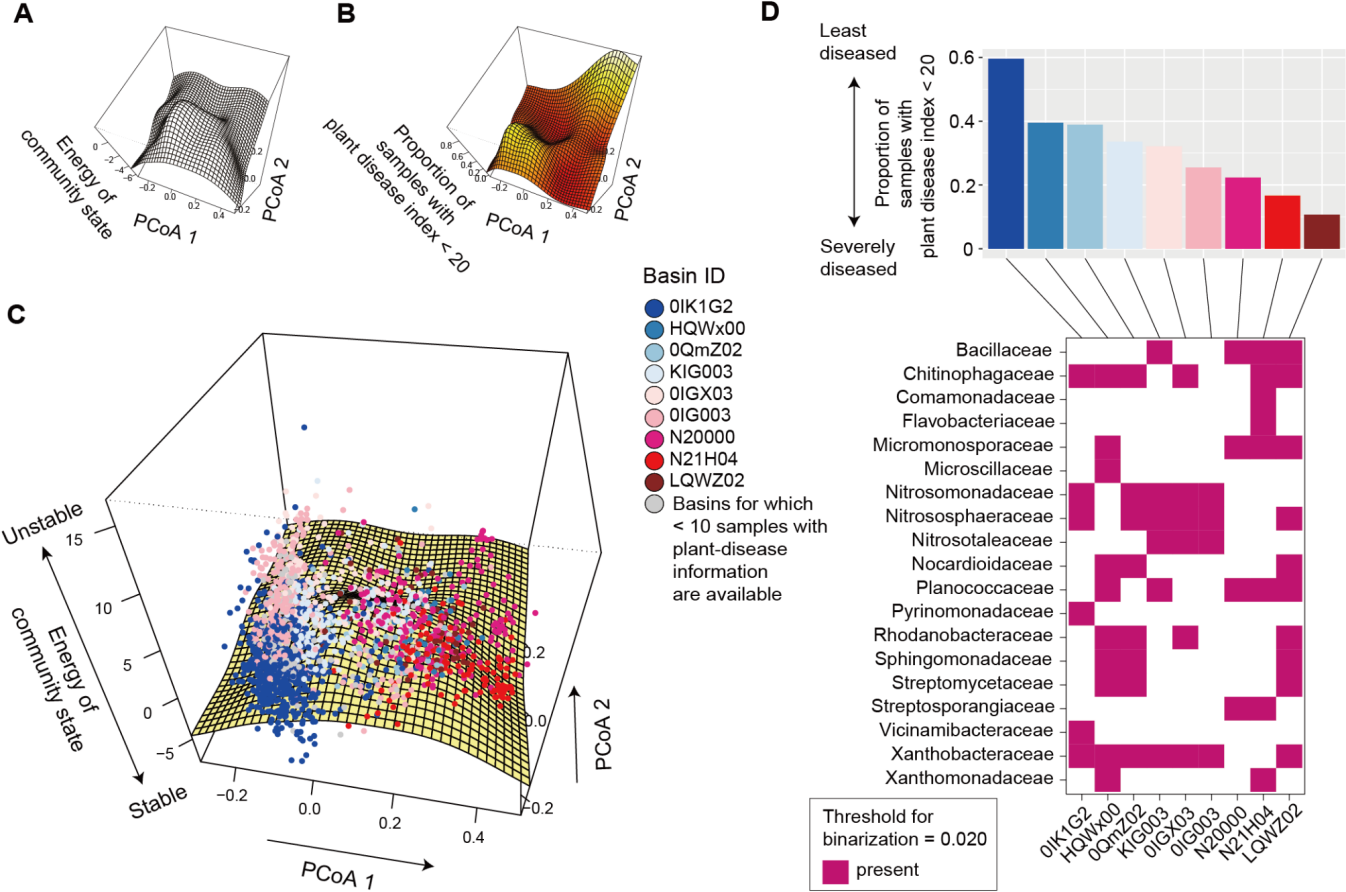
Energy landscape of prokaryotic communities. (**A**) Inferred energy landscape of family-level prokaryotic community structure (threshold for binarization = 0.020; occurrence threshold = 0.10; *S* = 35). The surface of energy levels was reconstructed across the PCoA space of fungal community structure (community PCoA1 and PCoA2 scores in Additional file2: Fig. S2) based on spline smoothing. Community states with lower energy are inferred to be more stable. (**B**) Landscape of crop disease prevalence. Across the PCoA space of prokaryotic compositions, the proportion of samples with disease severity index < 20 is shown based on spline smoothing. (**C**) Community data points on the energy landscape. The axis of “energy of community state” is more expanded than that in panel **A** in order to cover the range of samples. Data points (samples) indicated by the same color belong to the same basins of attraction, which are represented by IDs of the alternative stable states, whose energy is lower than that of any adjacent community states (i.e., bottoms of basins). (**D**) Key taxa whose abundance represent basins. In the upper panel, the mean proportion of soil samples with the minimum level of plant (crop) disease symptoms (the percentage of diseased plants < 20 or disease severity index < 20) is shown for each basin. The lower panel indicates the key taxa whose abundance characterizes difference among alternative stable states

**Fig. 5:**
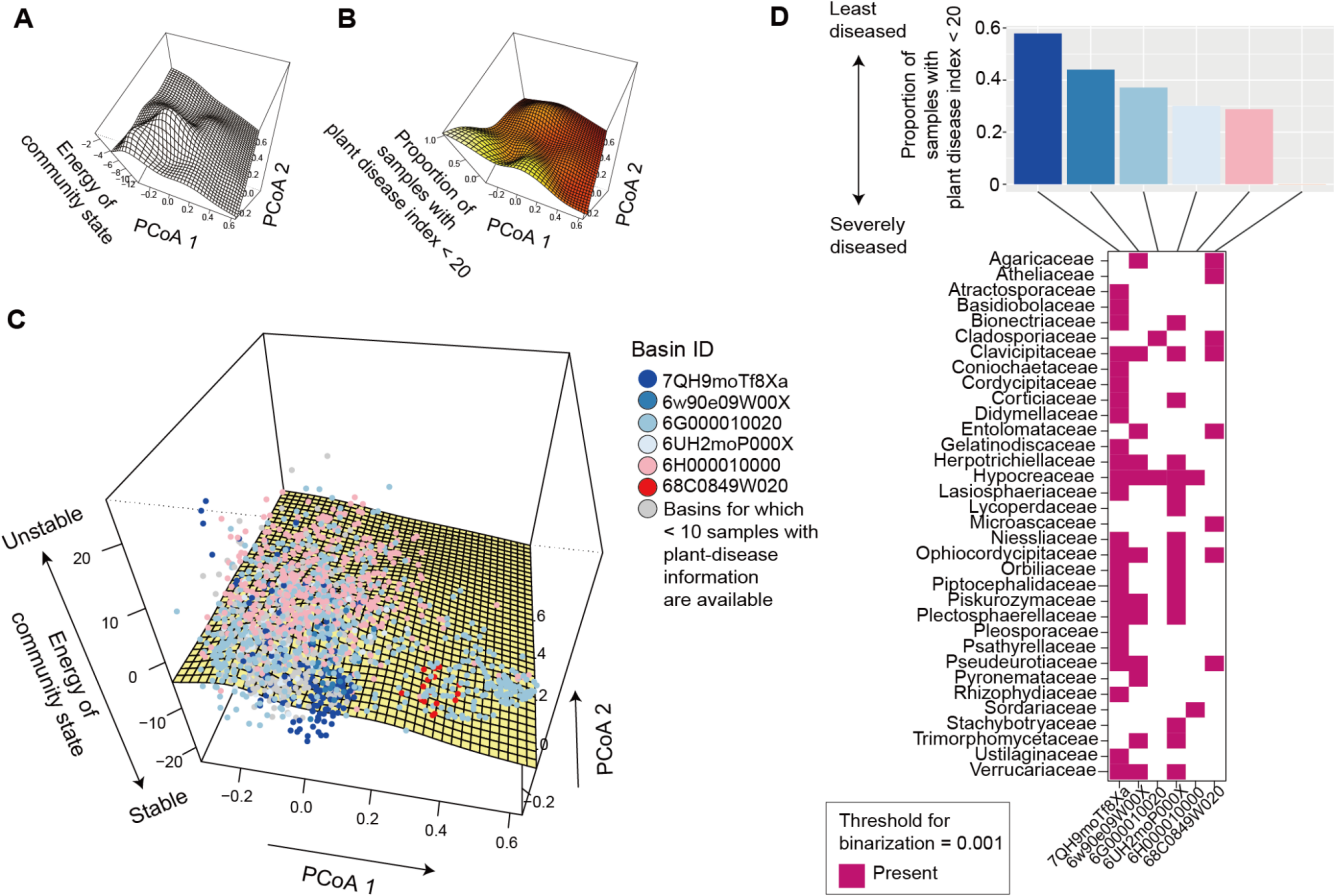
Energy landscape of fungal communities. (**A**) Inferred energy landscape of family-level fungal community structure (threshold for binarization = 0.001; occurrence threshold = 0.05; *S* = 62). The surface of energy levels was reconstructed across the PCoA space of fungal community structure (community PCoA1 and PCoA2 scores in Additional file 3: Fig. S3) based on spline smoothing. Community states with lower energy are inferred to be more stable. (**B**) Landscape of crop disease prevalence. Across the PCoA space of prokaryotic compositions, the proportion of samples with disease severity index < 20 is shown based on spline smoothing. (**C**) Community data points on the energy landscape. The axis of “energy of community state” is more expanded than that in panel **A** in order to cover the range of samples. Data points (samples) indicated by the same color belong to the same basins of attraction, which are represented by the IDs of alternative stable states, whose energy is lower than that of any adjacent community states (i.e., bottoms of basins). (**D**) Key taxa whose abundance represent basins. In the upper panel, the mean proportion of soil samples with the minimum level of plant (crop) disease symptoms (the percentage of diseased plants < 20 or disease severity index < 20) is shown for each basin. The lower panel indicates the key taxa whose abundance characterizes difference among the alternative stable states

The energy landscape of the family-level prokaryotic data included several major basins differing remarkably in associations with the prevalence of crop plant disease (Fig. 4). Specifically, among soil samples located within the basin represented by the alternative stable state 0IK1G2, 59.6% were associated with the lowest plant-disease level. Meanwhile, the proportion was only 10.7% for another basin (LQWZ02; Fig. 4C-D). The presence of basins differing greatly in their associations with plant-disease levels was inferred as well at the order-level analysis of the prokaryotic data (Additional file 4: Fig. S4). Such variation in crop disease prevalence among inferred basins was observed also for the family-level analysis of fungal community structure (Fig. 5). Specifically, while 57.9% of samples belonging to the basin 7QH9moTf8Xa, but none of the samples belonging to the basin 68C0849W020, were associated with the lowest plant-disease level (Fig. 5D). Meanwhile, such difference in associations with disease prevalence was moderate in an analysis in which a smaller number of fungal families were examined to define community states (Additional file 5: Fig. S5). The presence of multiple basins, which differed in associations with crop-disease prevalence, was suggested even when prokaryotic and fungal community data were simultaneously analyzed (Additional files 6–7: Figs. S6-7).

**Fig. 6:**
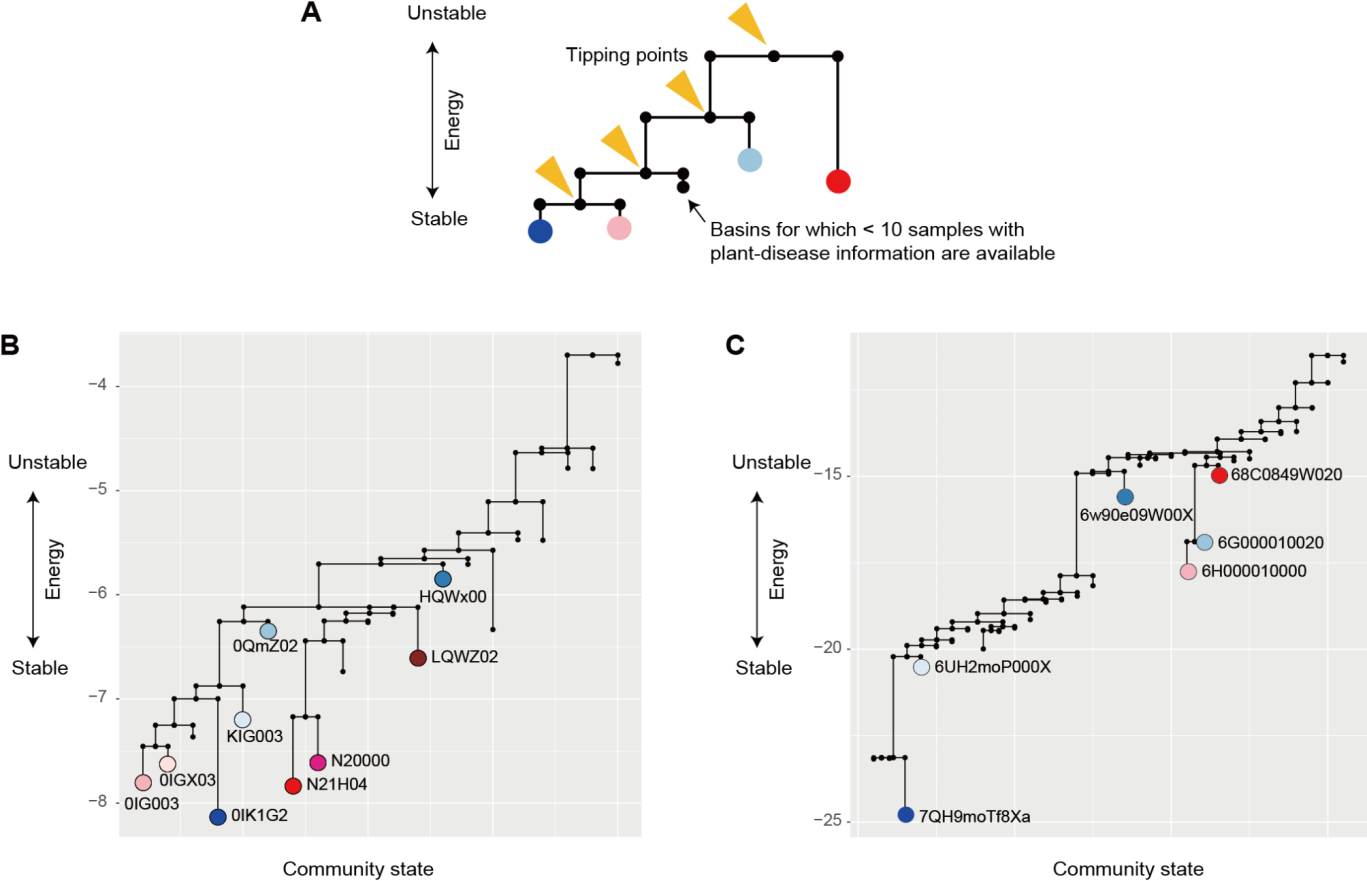
Disconnectivity graphs of the energy landscapes. (**A**) Schema of a disconnectivity graph. The energy of the “tipping points” splitting basins of attraction are presented across the axis of 2^*S*^ possible community states, where *S* denotes the number of the species or taxa examined. The energy of each alternative stable states is shown. (**B**) Tipping points and basins on the energy landscape of prokaryotes. The major basins of attraction with ≥ 10 samples with plant-disease information are highlighted with the colors defined in Fig. 4. (**C**) Tipping points and basins on the energy landscape of fungi. The major basins of attraction with ≥ 10 samples with plant-disease information are highlighted with the colors defined in Fig. 5

### Associations with crop disease level

In an analysis of the prokaryotic community structure, 19 families were keys to distinguish alternative stable states (or their representing basins) differing in associations with crop-disease prevalence (Fig. 4D). The presence of Pyrinomonadaceae and Vicinamibacteraceae, for example, was unique to the alternative stable state with the highest proportion of samples showing the lowest plant-disease level (Fig. 4D). Likewise, in an analysis of the fungal community structure, the alternative stable state associated closely with the lowest plant-disease prevalence (7QH9moTf8Xa) was defined by the presence of several families such as Basidiobolaceae, Cordycipitaceae, and Gelatinodiscaceae (Fig. 5D). The exploration of microbial taxa keys to distinguish basins with different ecosystem-level functions can be performed at other taxonomic levels (e.g., order-level; Additional files 4 and 7: Figs. S4 and S7).

### Disconnectivity graphs

Within the energy landscape of the family-level analysis of prokaryotes (Fig. 4), both the basins associated with the least-diseased (OIK1G2) and most-diseased (N21H04) crop status were the deepest among the inferred basins (i.e., showing the largest energy gaps from the bottom to tipping points; Fig. 6A-B). In the family-level analysis of fungi, the basin associated with the least-diseased status (7QH9moTf8Xa) was the deepest, while the other basin representing the most-diseased status (68C0849W020) was the shallowest (Fig. 6C).

## Discussion

We have estimated the stability landscape structure of complex soil microbiomes with the aid of a statistical framework commonly applicable to diverse types of biological communities. The energy landscape analysis allows systematic inference of community stability by integrating taxon-rich community datasets with the background information of multiple environmental factors [29, 34, 35]. While classic studies on community multistability have discussed ecological processes spanning a few intuitively distinguishable community states (high/low tree cover in forest–savanna transitions [9–11] or macrophyte-/phytoplankton-dominated state in shallow lakes [4, 8]), it is now made possible to define alternative stable states based on high-dimensional community datasets involving hundreds of species/taxa [16, 20–22, 44]. Such extension of discussion from simple to complex community characteristics is expected to deepen our understanding of alternative stable states in diverse microbial and macro-organismal systems.

Despite numerous potential compositions ($\:{2}^{S}$ community states; *S* is the number of considered species/taxa), the prokaryotic and fungal community states were grouped into small numbers of basins within the energy (stability) landscapes (Figs. 4 and 5). This result suggests that soil microbiome structure can remain within local regions (basins) even after demographic fluctuations (Fig. 1A). In other words, once trapped in a basin of attraction, large shifts in community structure would not occur without demographic perturbations whose strength exceed certain thresholds [1–3, 31]. Importantly, the threshold strength of perturbations is estimated as the energy gap between the bottoms of basins and tipping points [29] (Fig. 6A). Furthermore, potential paths of community structural transitions can be quantitatively inferred as illustrated in disconnectivity graphs [29] (Fig. 6B-C). Such statistical framework of quantitative science will entail novel opportunities for predicting abrupt shifts between alternative stable states in the era of high-throughput DNA sequencing, which provide massive data of ecological community compositions.

Among potential processes or mechanisms underlying the multistability of community structure, historical contingency is of particular interest [45]. In the local assembly of microbial communities, early colonizers or residents can prevent the settlement of followers by constructing physical barriers (e.g., biofilms and mycelia) [45–49] or emitting antibiotics [50, 51]. In addition to those antagonistic effects on late colonizers, webs of mutualistic or commensalistic interactions within the microbiomes of early colonizers [52–54] would influence community dynamics. Due to such “priority effects” [45], bacterial and fungal community compositions may persist within limited ranges of community states without substantial perturbations. Given that abilities to form physical or chemical barriers can differ greatly among microbial species/taxa [47, 50, 51], such variation in constituent species’ priority effects may underly the observed variation in the depth of basins (Fig. 6B-C).

The inference of stability landscape structure provided an opportunity for evaluating relationship between community stability and ecosystem-scale functions. The alternative stable states of prokaryotic/fungal community structure differed considerably in associations with crop disease prevalence (Fig. 5), suggesting the presence of “stable and favorable” and “stable but unfavorable” states of microbiomes [55–57] in terms of agricultural productivity. This finding adds an important dimension of discussion on the use of microbes in agriculture. Beyond investigations on single species/strains of microbes, microbiome studies have explored sets of microbes that collectively maximize biological functions [15, 58–60]. In particular, experimental studies on “synthetic” communities have reorganized our knowledge of microbiome functions [58–60]. Nonetheless, such microbial functions cannot be realized in real agroecosystems if the synthesized or designed microbiome compositions are vulnerable to biotic and abiotic environmental changes in the wild [61]. Thus, in addition to functional properties, compositional stability is the key to manage microbiomes in agroecosystems [58, 62, 63].

In our analysis across the Japan Archipelago, prokaryotic and fungal taxa keys to distinguish least-diseased and severely-diseased states of soil microbiomes were highlighted (Figs. 4 and 5). Among them, Basidiobolaceae and Cordycipitaceae are of particular interest because they include many species potentially utilized as biological control agents for suppressing pest insects [64, 65]. Gelatinodiscaceae is another fungal taxon playing potentially important roles as symbionts of plants [66]. These results illuminate the hypothesis that plant disease could be suppressed under the coexistence of multiple prokaryotic and fungal taxa with favorable ecosystem functions [15, 67]. Thus, statistical analyses of stability landscapes allow the exploration of key species or taxa [68, 69], whose management could result in transitions from unfavorable ecosystem states to favorable ones [2, 4, 12, 13]. Given that most prokaryotic and fungal families highlighted in our analysis have cosmopolitan distributions, a next crucial step is to test whether the alternative stable states defined across the Japan Archipelago can be used to categorize disease-suppressive and disease-susceptible microbiomes in other regions on the globe.

Although the energy landscape analysis enhances our understanding of community stability and functions, its results should be interpreted with caution. First, given that classic ecological studies tended to examine community multistability with system-specific simple criteria (e.g., high/low tree cover [9–11]), special care should be taken when we extend the existing theoretical literature to the studies on species-rich (high-dimensional) community data [44]. In other words, unambiguous and broadly applicable criteria based on statistical evaluation are the prerequisite for comparative analyses of community multistability. Although we applied a straightforward statistical definition of alternative stable states [29] (Fig. 1) by taking into account classic theoretical concepts [1–3, 31], continuous methodological improvements should be explored towards further comprehensive understanding. Second, our analysis on hyper-diverse soil microbiomes incurred substantial computational costs, forcing us to limit the energy landscape analysis to family-level input data. Further improvements of codes are necessary for inferring stability landscapes at genus-, species-, or strain-level analyses. Third, it should be acknowledged that detailed discussion on ecological processes require time-series datasets [70–72]. Because our present data lacked the information of temporal changes in community structure, we are unable to discuss the frequency and pace of community structural transitions between alternative stable states. Monitoring of microbiome compositions [19, 21, 27] is necessary for filling the gap between theoretical and empirical studies [73].

## Conclusion

As shown in this study, the relationship among community structure, stability, and functions can be overviewed based on a statistical framework of the energy landscape analysis. The application of the analysis to a large DNA-sequencing-based dataset suggested that agroecosystem soil microbiomes could be classified into several compositional groups representing alternative stable states. We then found that the inferred alternative stable states differed greatly in their associations with ecosystem-level properties (crop-disease levels). The energy landscape analysis then allowed us to consider potential paths of transitions between the alternative stable states. Consequently, the statistical-physics-based approach is expected to fill the gap between classic ecological theory and empirical microbiome research by extending the targets of discussion on the multistability of ecological communities.

The energy landscape framework of multistability analysis is readily applicable to a wide range of microbiome datasets. Application to human microbiome data is of particular interest in terms of the confirmation of the existence of multiple basins of attraction [25]. In addition, insights into the key microbial species/taxa that would play key roles in the transitions from disease-associated microbiome states to healthy ones will open new directions of microbiome therapy. Furthermore, time-series analyses of community dynamics on stability landscapes will allow us to forecast transitions into unfavorable community states (e.g., dysbiosis [20, 73, 74]). In line with such proof-of-concept research targeting human microbiomes, the application of the statistical framework to environmental microbiome data will deepen our understanding of the multistability of aquatic and terrestrial ecosystems. Meanwhile, the current version of the energy landscape analysis has not been designed to clarify causality among environmental conditions, community structure, and ecosystem-scale properties. In general, causality between variables cannot be detected from observational data based on standard statistical approaches. Thus, further methodological improvements are required to build a general platform for exploring key driving factors of microbiome dynamics. Along with such extensions of observational approach, experimental studies controlling key biotic or abiotic environmental parameters [6] will promote both basic and applied sciences of ecosystem functions, fueling research on ecosystem restoration and sustainable agroecosystem management.

## Electronic supplementary material

Below is the link to the electronic supplementary material.

Supplementary Material 1

Supplementary Material 2

Supplementary Material 3

Supplementary Material 4

Supplementary Material 5

Supplementary Material 6

Supplementary Material 7

## Data Availability

The accession number of the DDBJ Sequence Read Archive: DRA015491. The microbial community matrices are provided with computer codes at our GitHub repository (https://github.com/hiro-toju/Soil_EnergyLandscape_NARO3000). The tutorials and codes of energy landscape analyses are available at https://github.com/kecosz/rELA.

## Abbreviations

ASV: Amplicon sequence variant
C/N: Carbon/nitrogen
DDBJ: DNA Data Bank of Japan
ITS1: Internal transcribed spacer 1
PCoA: Principal coordinate analysis
