# Supplementary material for: Alternative stable states of microbiome structure and soil ecosystem functions
Source: Environ Microbiome. 2025 Mar 6;20:28. doi: 10.1186/s40793-025-00688-4 (PMC11887376; doi:10.1186/s40793-025-00688-4)
Supplement: Supplementary file 1 — Supplementary Material 1 [file 40793_2025_688_MOESM1_ESM.pdf]

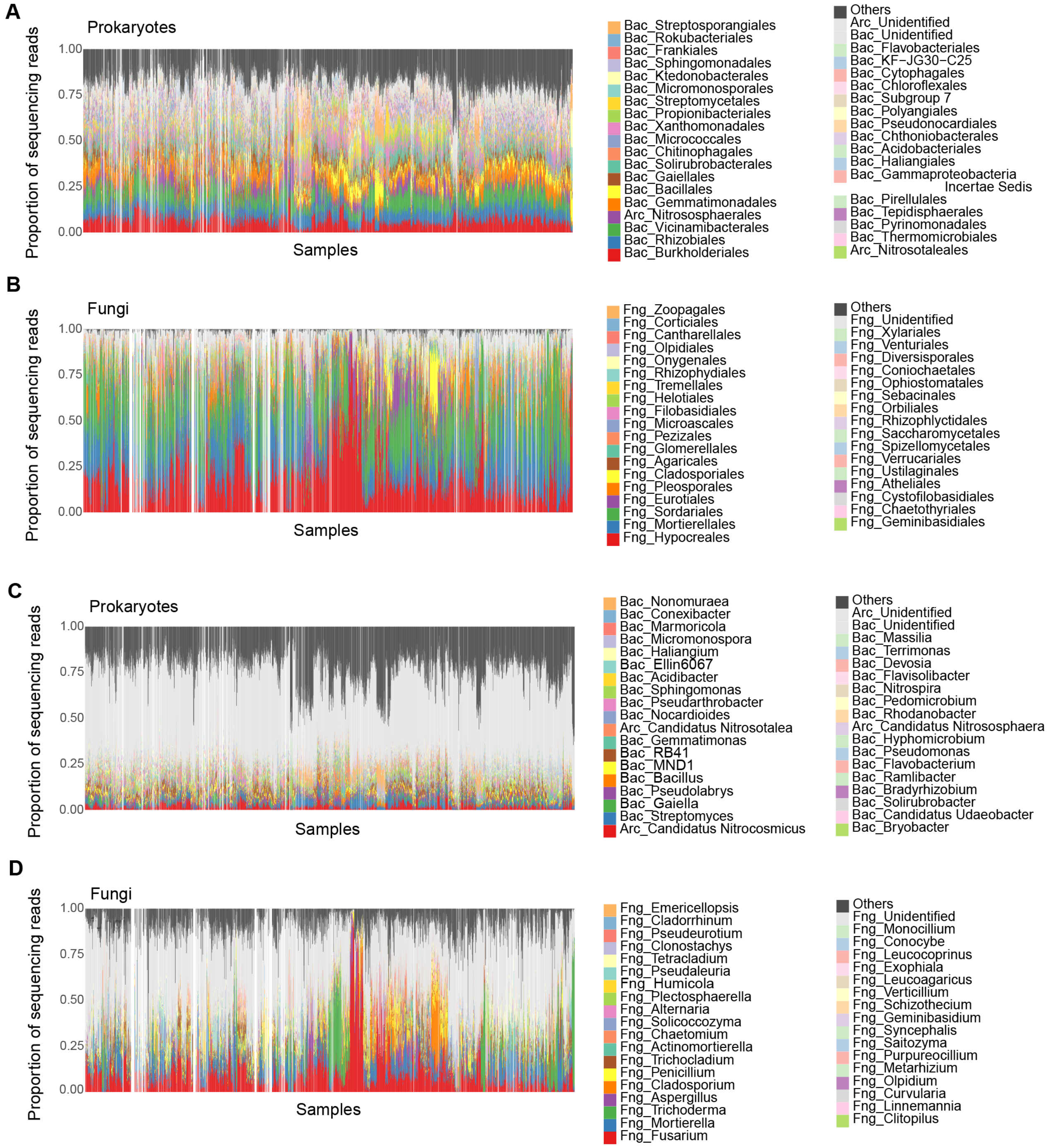

Additional file 1: Fig. S1. Community structure of the source data (order- and genus-level compositions). (**A**) Order-level compositions of prokaryotic communities. (**B**) Order-level compositions of fungal communities. (**C**) Genus-level compositions of prokaryotic communities. (**D**) Genus-level compositions of fungal communities. The soil samples from which DNA sequence data were unavailable for either prokaryotic 16S rRNA or fungal ITS regions are indicated as blanks.
