# Supplementary material for: Alternative stable states of microbiome structure and soil ecosystem functions
Source: Environ Microbiome. 2025 Mar 6;20:28. doi: 10.1186/s40793-025-00688-4 (PMC11887376; doi:10.1186/s40793-025-00688-4)
Supplement: Supplementary file 2 — Supplementary Material 2 [file 40793_2025_688_MOESM2_ESM.pdf]

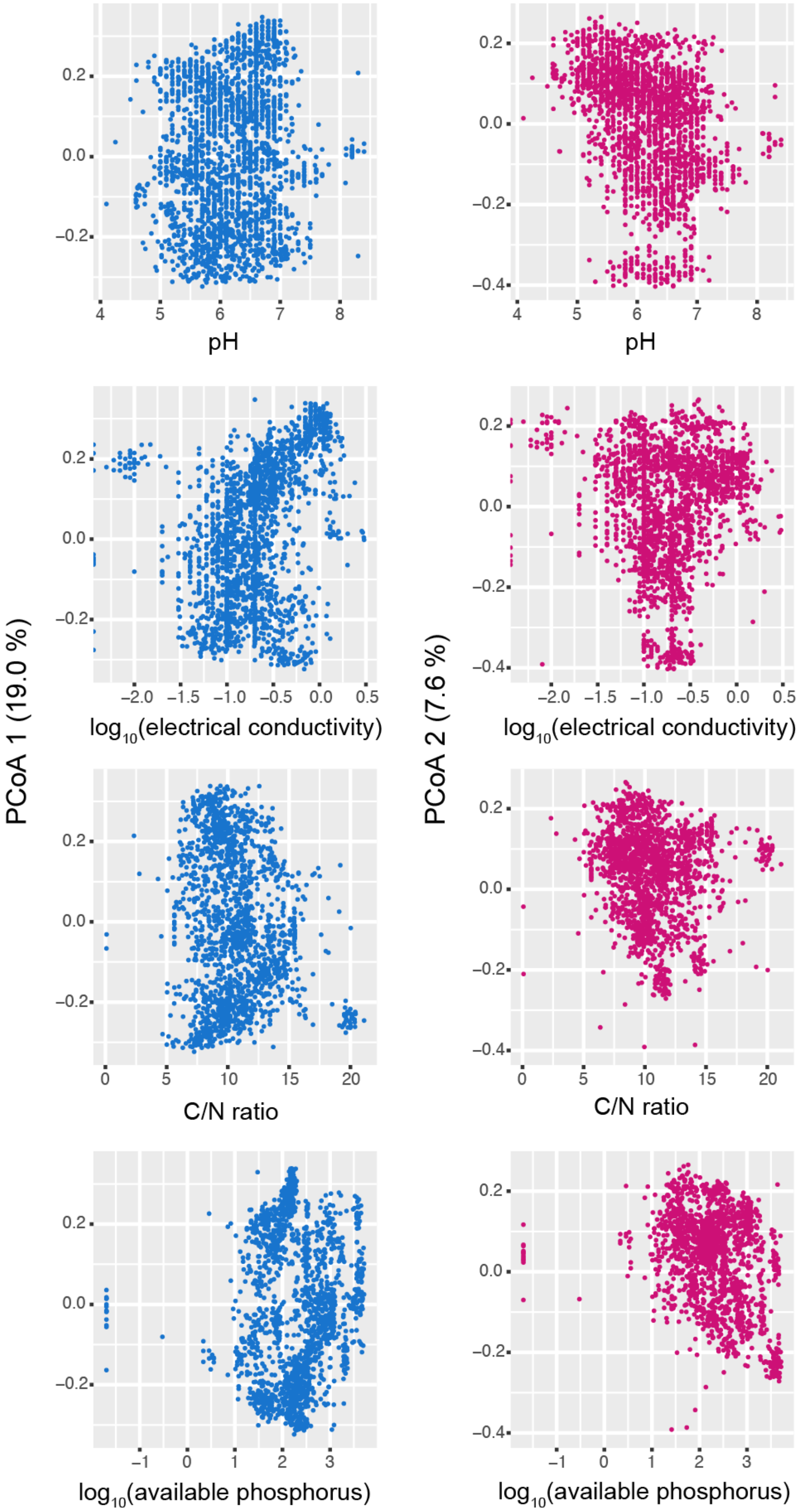

Additional file 2: Fig. S2. Prokaryotic community structure along environmental gradients (scatter plots). The scores representing prokaryotic community compositions (PCoA 1 and 2 scores) are shown along each axis of soil environmental factors (pH, electrical conductivity, C/N ratio, and available phosphorus concentration). See Figure 3 for density plots.
