# Supplementary material for: Alternative stable states of microbiome structure and soil ecosystem functions
Source: Environ Microbiome. 2025 Mar 6;20:28. doi: 10.1186/s40793-025-00688-4 (PMC11887376; doi:10.1186/s40793-025-00688-4)
Supplement: Supplementary file 4 — Supplementary Material 4 [file 40793_2025_688_MOESM4_ESM.pdf]

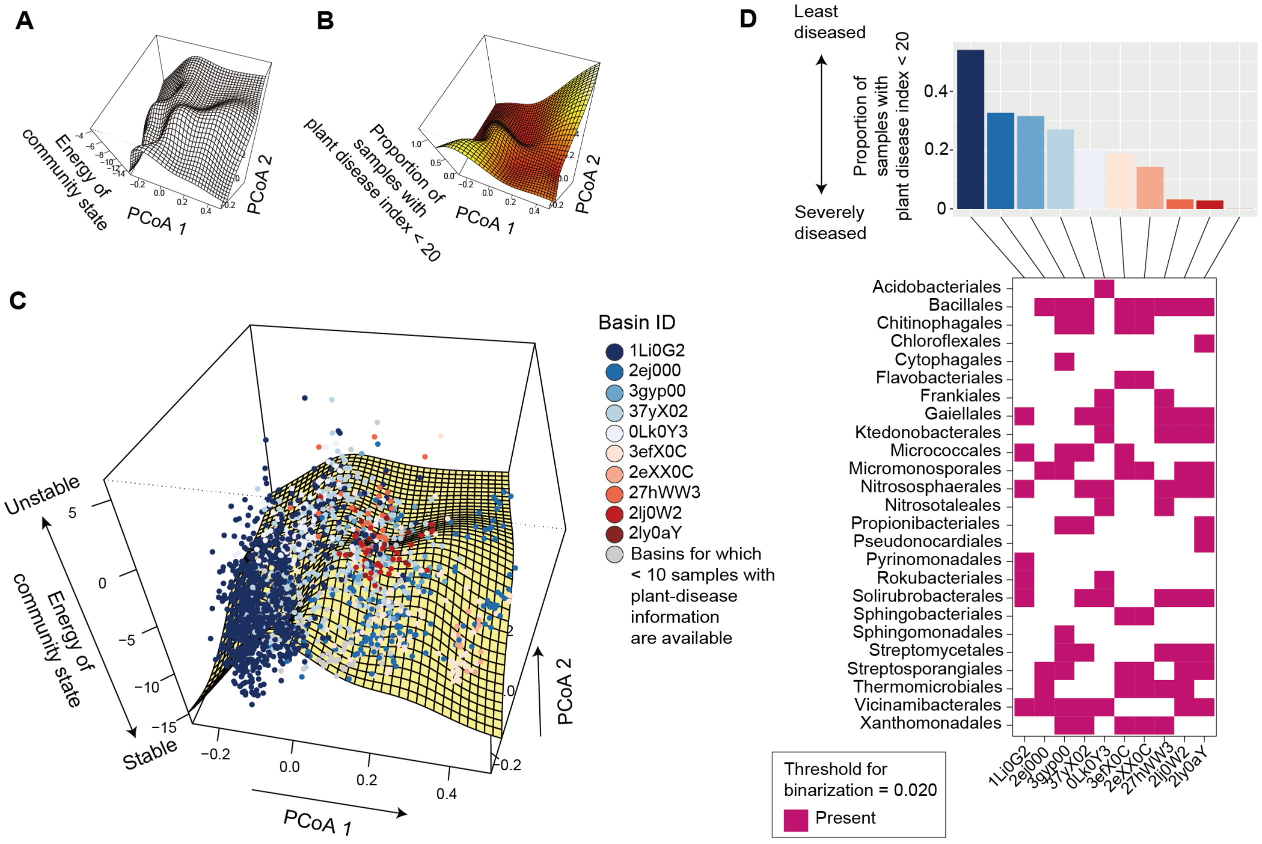

Additional file 4: Fig. S4. Energy landscape of prokaryotic communities (order-level compositions; threshold for binarization = 0.020; occurrence threshold = 0.10; S = 32). (**A**) Inferred energy landscape of order-level prokaryotic community structure. The surface of energy levels was reconstructed across the PCoA space of fungal community structure (community PCoA1 and PCoA2 scores in in Figures 2–figure supplement 1) based on spline smoothing. Community states with lower energy are inferred to be more stable. (**B**) Landscape of crop disease prevalence. Across the PCoA space of prokaryotic compositions, the proportion of samples with disease severity index < 20 is shown based on spline smoothing. (**C**) Community data points on the energy landscape. The axis of "energy of community state" is more expanded than that in panel **A** in order to cover the range of samples. Data points (samples) indicated by the same color belong to the same basins of attraction, which are represented by the IDs of alternative stable states, whose energy is lower than that of any adjacent community states (i.e., bottoms of basins). (**D**) Key taxa whose abundance represent basins. In the upper panel, the mean proportion of soil samples with the minimum level of plant (crop) disease symptoms (the percentage of diseased plants < 20 or disease severity index < 20) is shown for each basin. The lower panel indicates the key taxa whose abundance characterizes difference among the alternative stable states.
